# How can health ministries present persuasive investment plans for women’s, children’s and adolescents’ health?

**DOI:** 10.2471/BLT.15.168419

**Published:** 2016-05-01

**Authors:** Ian Anderson, Blerta Maliqi, Henrik Axelson, Mikael Ostergren

**Affiliations:** a Crawford School of Public Policy, Australian National University, 132 Lennox Crossing, Acton, Canberra, 0200, Australia.; b Department of Maternal, Newborn, Child and Adolescent Health, World Health Organization, Geneva, Switzerland.; c Independent consultant, Geneva, Switzerland.

## Abstract

Most low- and middle-income countries face financing pressures if they are to adequately address the recommendations of the Global Strategy for Women’s, Children’s and Adolescent’s Health. Negotiations between government ministries of health and finance are a key determinant of the level and effectiveness of public expenditure in the health sector. Yet ministries of health in low- and middle-income countries do not always have a good record in obtaining additional resources from key decision-making institutions. This is despite the strong evidence about the affordability and cost–effectiveness of many public health interventions and of the economic returns of investing in health. This article sets out 10 attributes of effective budget requests that can address the analytical needs and perspectives of ministries of finance and other financial decision-makers. We developed the list based on accepted economic principles, a literature review and a workshop in June 2015 involving government officials and other key stakeholders from low- and middle-income countries. The aim is to support ministries of health to present a more strategic and compelling plan for investments in the health of women, children and adolescents.

## Résumé

La plupart des pays à revenu faible et intermédiaire font face à des contraintes de financement pour pouvoir suivre correctement les recommandations de la Stratégie mondiale pour la santé de la femme, de l'enfant et de l'adolescent. Les négociations entre le ministère de la Santé et celui des Finances sont un déterminant clé de l'importance et de l'efficacité des dépenses publiques en matière de santé. Or, dans les pays à revenu faible et intermédiaire, le ministère de la Santé ne parvient pas toujours à obtenir des ressources supplémentaires de la part des grandes institutions décisionnaires, en dépit d'éléments démontrant l'abordabilité et la rentabilité de nombreuses actions de santé publique et du rendement économique des investissements dans le domaine de la santé. Cet article présente 10 caractéristiques convaincantes de demandes de crédits permettant de tenir compte des besoins analytiques et des perspectives des ministères des Finances et d'autres décisionnaires financiers. Nous avons établi cette liste à partir des principes économiques courants, d'une analyse documentaire et d'un atelier organisé en juin 2015, auquel ont participé des représentants de gouvernements et d'autres acteurs clés de pays à revenu faible et intermédiaire. L'objectif est d'aider les ministères de la Santé à présenter un plan d'investissement plus stratégique et plus convaincant dans le domaine de la santé de la femme, de l'enfant et de l'adolescent.

## Resumen

La mayoría de los países con ingresos bajos y medios afrontan presiones de financiación para poder abordar de forma adecuada las recomendaciones de la Estrategia Mundial para la Salud de la Mujer, el Niño y el Adolescente. Las negociaciones entre los ministerios de sanidad y economía son un determinante fundamental del nivel y eficacia del gasto público en el sector sanitario. Aun así, los ministerios de salud de países con ingresos bajos y medios no siempre cuentan con una buena trayectoria a la hora de obtener recursos adicionales de instituciones fundamentales para la toma de decisiones. Esto se produce a pesar de la fuerte prueba acerca de la asequibilidad y rentabilidad de muchas intervenciones de salud pública y de los beneficios económicos de la inversión en sanidad. Este artículo presenta 10 características de solicitudes presupuestarias eficaces que pueden abordar las necesidades analíticas y las perspectivas de los ministerios de economía y otros responsables de la toma de decisiones financieras. La lista se desarrolló en base a los principios económicos aceptados, una revisión bibliográfica y un taller realizado en junio de 2015 en el que participaron funcionarios del gobierno y otras partes interesadas fundamentales de países con ingresos bajos y medios. El objetivo es dar apoyo a los ministerios de salud para que presenten un plan más estratégico y completo para invertir en la salud de mujeres, niños y adolescentes.

## ملخص

سوف تواجه أغلب البلدان منخفضة ومتوسطة الدخل ضغوطًا للتمويل إذا تناولت توصيات الاستراتيجيات العالمية حول صحة المرأة والطفل والمراهقين بشكل كافٍ. وتمثل المفاوضات بين الوزارات الحكومية للصحة والمالية المحدد الرئيسي لمستوى الإنفاق العام في قطاع الصحة ومدى فعاليته، إلا أن وزارات الصحة في البلدان منخفضة ومتوسطة الدخل تفتقر دائمًا إلى سجل مشهود يشير لنجاحها في الحصول على موارد إضافية من مؤسسات صنع القرارات الرئيسية. وهذا على الرغم من الأدلة القوية عن معقولية الأسعار وفعالية تكلفة العديد من التدخلات الصحية العامة والعوائد الاقتصادية للاستثمار في الصحة. تنص هذه المقالة على 10 سمات لطلبات الميزانية الفعالة التي يمكنها أن تتناول الاحتياجات التحليلية ووجهات نظر وزارات المالية وصناع القرار الماليين الآخرين. وقد قمنا بوضع القائمة بناءً على المبادئ الاقتصادية المقبولة، واستعراض الكتابات، وحلقة العمل التي انعقدت في يونيو/حزيران 2015 والتي شارك فيها مسؤولون حكوميون وجهات معنية رئيسية أخرى من البلدان منخفضة ومتوسطة الأجر. ويتمثل الهدف منها في دعم وزارات الصحة لتقديم خطة أكثر استراتيجية ومقنعة للاستثمارات في صحة المرأة والطفل والمراهقين.

## 摘要

对于大多数低收入和中等收入国家来说，要想充分落实《妇女、儿童和青少年健康全球战略》的建议，各国面临着融资压力。政府卫生部和财政部之间的协商是公共支出在卫生领域支出水平和效率的关键因素。然而从过往经验来说，低收入和中等收入国家的卫生部并不总是能够从关键的决策机构那里获得额外资源。尽管在负担能力和成本效益方面有足够的证据证明许多公共卫生干预措施有效且卫生投资的经济回报率颇高。本文针对财政部和其他财政决策者的分析需求和看法，列出了有效预算申请的 10 个特性。我们依据公认的经济原则、文献综述以及 2015 年 7 月由许多低收入和中等收入国家政府官员和其他关键利益相关者参与的研讨会制定了列表。此举的目的是为了支持卫生部针对妇女、儿童以及青少年的健康提出更具战略性和信服力的投资计划。

## Резюме

Большинство стран с низким и средним уровнем доходов испытывают финансовые трудности при попытке надлежащего воплощения в жизнь рекомендаций Глобальной стратегии по здоровью женщин, детей и подростков. Переговоры между министерствами здравоохранения и финансов являются ключевым фактором, определяющим уровень и эффективность государственных расходов в сфере здравоохранения. Тем не менее министерствам здравоохранения в странах с низким и средним уровнем доходов не всегда удается получить дополнительные ресурсы у основных организаций, принимающих решения. Такое возможно даже при наличии веского обоснования осуществимости и эффективности инвестиционных мер многих государственных мероприятий в области здравоохранения, а также будущей экономической отдачи такого инвестирования. В данной статье перечисляются 10 характерных признаков убедительных бюджетных запросов, в которых учитывались бы потребности и интересы министерств финансов и других лиц, принимающих решения в этой области, в части анализов, проводимых ими. На основе принятых экономических принципов был составлен список таких признаков. Кроме того, в качестве материалов для статьи были использованы обзор литературных источников и результаты практикума, проведенного в июне 2015 года, в котором приняли участие государственные должностные лица и другие ключевые участники из стран с низким и средним уровнем доходов. Целью данной статьи является содействие министерствам здравоохранения в представлении более продуманного и убедительного плана инвестиций в сферу охраны здоровья женщин, детей и подростков.

## Introduction

Most low- and middle-income countries face financing pressures if they are to adequately address global goals for improving the health of women, children and adolescents. Only six of the 75 priority countries achieved the 5.5% annual rate of reduction in maternal mortality needed to achieve the United Nations (UN) millennium development goal 5.^1^ Now further investments are required to meet the sustainable development goals for 2030 and implement the recommendations in the updated UN Global Strategy for Women’s, Children’s and Adolescents’ Health (2016–2030).^2^ According to the strategy, “existing financing falls far short of the sums needed to fund all the measures envisioned in this strategy. To scale up from current coverage to the targets for 2030 requires 33.3 billion United States dollars (US$) in 2015 alone across 63 high-burden, low-income and lower-middle-income countries, equivalent to US$ 10 per capita”.

**Fig. 1:**
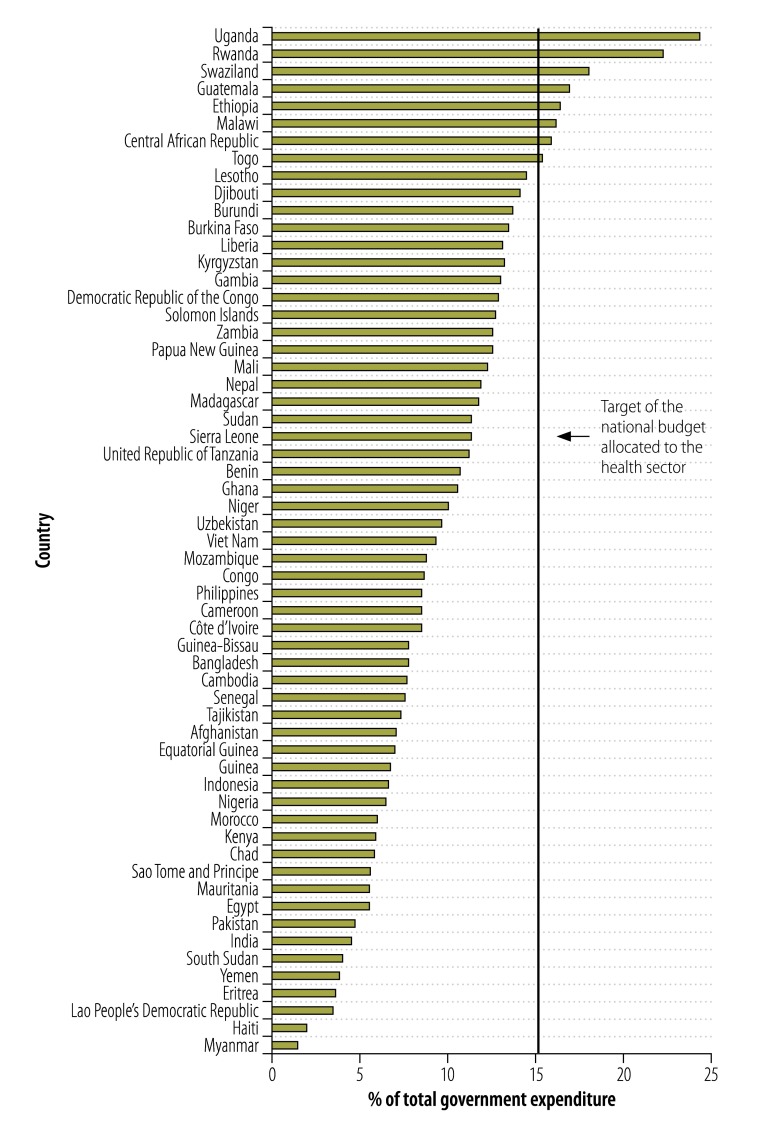
Public health expenditure as a percentage of total government expenditure in countries eligible for financing from the global financing facility for women and children’s health, 2013

Note: Target agreed to by many low-income governments in 2001.^6^

How much low- and middle-income countries spend from their own resources, and how well they spend them, is a key determinant of the outputs and outcomes of women’s, children’s and adolescents’ health. There is no consensus about the amount or proportion of national income to spend on health;^3^ good outcomes for maternal health, for example, can be achieved even in low-income settings.^4^ Nevertheless, government expenditure on health is often low in absolute and relative terms in many low- and middle-income countries. This is important, because governments are usually the principal provider of public goods – worthwhile interventions that the private sector tend not to supply because they cannot easily charge a price for it, such as disease surveillance, vector control and other public-wide interventions against infectious diseases. Government expenditure is also a potentially key instrument for addressing health-related poverty and inequity and for preventing impoverishment due to out-of-pocket private expenditure. Sixty-three high-burden, low- and lower-middle-income countries are eligible for support under the recently launched global financing facility in support of the UN Secretary-General’s Every Woman Every Child global strategy.^2^ Yet in 48 of these countries the government’s expenditure on health in 2013 was less than US$ 50 per capita, with eight countries spending less than US$ 10 per capita, and as low as US$ 4 per capita in Myanmar.^5^ Fig. 1 shows that only nine countries have achieved the target of allocating 15% of national budget to the health sector which was agreed to by many low-income governments in 2001.^6^ Eight countries, including highly populated India and Pakistan, allocated less than 5% of total government expenditure to health.

Data source: Global health observatory data repository.^7^

The share of government expenditure being allocated to women’s and children’s health, and the health sector more broadly, is also important because it reveals the political priority given to health compared with other sectors and priorities. An analysis of national health accounts for this article showed that the one lower-middle income and nine low-income countries for which data were available allocated on average just 22.6% of government health expenditure to reproductive, maternal, newborn and child health (expenditure on adolescents was not captured), despite that group normally comprising more than 50% of the population.

Governments in low- and middle-income countries already spend on average US$ 19.8 of their own resources on health for every dollar they receive in external assistance,^8^ but virtually all such countries face significant pressures for additional government expenditure in coming years. Population growth will put additional demands on already under-funded public health systems.^9^ Almost all low- and middle-income countries are seeking to achieve universal health coverage, yet most are also experiencing increased financial pressures as a result of rising rates of often expensive-to-treat and chronic noncommunicable diseases. Many countries are losing access to external development finance as they move into middle-income status.^8^ At the same time, there is increasing competition for those requiring aid financing for health. How well ministries of health can negotiate with ministries of finance to access and then spend additional financing is therefore a key issue.

## Negotiations between ministries

Many factors, especially political economy factors, affect the level and allocation of public resources for health. Different stakeholders – including government institutions, politicians, development partners, the private sector, civil society and individual households – also ultimately influence the level and allocation of expenditure on women’s, children’s and adolescents’ health. However, the interaction between the ministry of health and ministry of finance is critical for priority setting and resource allocation decisions. The ministry of health is key to this because it is the institution primarily responsible for advocating additional public financial resources and then managing public expenditure. The ministry of finance is also key because it is usually the most influential institution helping to decide the allocation of resources among competing needs and sectors. All too often health ministry officials are unable to convince their finance ministry counterparts that additional public expenditure is justified. This is despite the fact that the health sector has a strong evidence base, including randomized control trials, to demonstrate the affordability and cost–effectiveness of interventions, immunization being just one example. There can be missed opportunities to successfully negotiate additional financing due to the way in which health ministries approach finance ministries. The ministry of health proposal may have the appearance of an ad hoc shopping list rather than genuine investment plan that demonstrates prioritized use of scarce resources with a clear focus on significant results and value for money.

Against that background, we have identified 10 key attributes that ministries of finance and other financial decision-makers will normally be looking for in any investment plan being submitted from the ministry of health (Box 1). We developed the list based on accepted economic principles, a literature review with key search terms including “priority setting in health”, “resource allocation in health”, “public expenditure for health” and “low and middle income countries”. We also explored the issues during a two-day workshop entitled *From shopping lists to investment plans* held in June 2015 in Geneva, Switzerland, involving 48 government officials and other key stakeholders from ministries of health and finance, from low- and middle-income countries, as well as several bilateral and multilateral development partners.^10^

Box 1 **Ten attributes of an effective health ministry investment plan to ministry of finance decision-makers**

1. Demonstrates how health programmes contribute to broader national development objectives
2. Explains and quantifies how well-designed and well-targeted health expenditure is an investment not merely a cost
3. Demonstrates good use of existing financial and other resources by the health sector
4. Demonstrates effective allocation of existing health sector resources, with a focus on results
5. Shows how health expenditure is cost–effective and even cost-saving to government, development partners and households
6. Identifies and explains market failures in health provision that require public expenditure
7. Identifies and quantifies mutual benefits for the public health and finance sectors: improving health while raising additional government revenue
8. Presents a clear and accountable plan for downstream implementation, management, evaluation and lesson-learning from health programmes
9. Presents a strong evidence base for health policy and programming decision-making
10. Avoids earmarking of funds to the health sector, but shows how investment in the health sector complements investments in other sectors such as education

## Key funding attributes

A starting point for the ministry of finance will be how the ministry of health’s proposals can specifically contribute to broader national development objectives. Improving health and saving lives has, of course, intrinsic value. However, the health ministry needs to also specifically demonstrate how, where, when and at what cost investments in health directly contribute to broader priority national objectives and not just to health goals, important as they may be. There are many arguments that the ministry of health can make, depending on the country. For example, investments in health, including family planning, have been shown to contribute directly and indirectly to favourable demographic trends, better learning outcomes, higher worker productivity and greater household savings and investment, and therefore to better longer term economic growth, often at low per capita cost.^11^^–^^16^ Box 2 gives examples of investments in women’s, children’s and adolescents’ health that contribute directly to economic growth and poverty reduction.

Box 2 **Examples of how investment in women’s, children’s and adolescents’ health contributes directly to economic growth and poverty reduction**

- Recent analysis of the Global Investment Framework for Women’s and Children’s Health suggests increasing health expenditure by just US$ 5 per person per year up to 2035 in 74 high-burden countries could yield up to nine times that value in economic and social benefits.^6^
- An estimated 30–50% of eastern Asia’s dramatic economic growth in 1965–1990 is attributed to reduced child mortality and subsequent lower fertility rates that created a baby-boom cohort and decreased the dependency ratio.^17^
- Good access to affordable and quality health-care significantly reduces catastrophic out-of-pocket health care costs that push, or keep, millions of people below the poverty line worldwide, undermining nations’ poverty alleviation goals.^18^
- Shifting from the UN medium-fertility population projection to the UN low-fertility population projection is estimated to raise income per capita by 5.6% over 20 years, and by 11.9% over 50 years.^19^

Second, the ministry of finance will usually want to see that the proposed expenditure is an investment yielding substantial outputs and outcomes, and not simply a cost, with a focus on inputs and expenses. Proposals that can demonstrate measurable outputs and outcomes in ways that are affordable, feasible, financially and institutionally sustainable, cost–effective or even potentially cost-saving, and yield economic returns on investment in a wide range of settings are more convincing than budget proposals that focus just on inputs. Box 3. provides examples of effective investment for women’s, children’s and adolescents’ health.

Box 3 **Examples of how investment in women’s, children’s and adolescents’ health can yield significant outcomes at low cost**

If coverage of key evidence-based interventions were scaled up to at least 80%, and immunization coverage to at least 90%, 95% of deaths due to diarrhoea and 67% due to pneumonia in children younger than 5 years could be eliminated by 2025 at an additional cost globally of US$ 2.9 billion.^20^

Sri Lanka has been able to halve maternal deaths relative to the number of live births every 6 to 12 years for many decades since 1935, despite having spent less on health than most countries of similar income level.^21^

Third, a ministry of finance will be more confident about allocating additional resources if the ministry of health provides evidence that it is already making good use of its existing resources. A finance ministry may concede that the health ministry requires additional funding, but may be reluctant to allocate additional funds if it knows or perceives, for example, that public health facilities are irregularly staffed or underused; that there has been under-expenditure in the health budget in previous years; or that there are inefficiencies in procurement or cases of waste and corruption. The World Health Organization (WHO) notes that around 20–40% of health expenditure globally could be freed up through eliminating 10 preventable sources of waste and inefficiency in the health sector (Box 4).^22^ A World Bank report estimated that in Cambodia savings could exceed US$ 50 million a year or one-third of government health spending (the equivalent of 0.4% of gross domestic product), through more efficient purchasing of pharmaceuticals, medical equipment and supplies.^23^

Box 4 **Ten leading sources of inefficiency in health systems**

**Medicines**

- Underuse of generics and higher than necessary prices for medicines
- Use of substandard and counterfeit medicines
- Inappropriate and ineffective use

**Health-care services**

- Overuse or undersupply of equipment, investigations and procedures
- Inappropriate hospital admissions and length of stay
- Inappropriate hospital size (low use of infrastructure)
- Medical errors and suboptimal quality of care

**Broader health system**

- Health workers: inappropriate or costly staff mix, unmotivated workers
- Health system leakages: waste, corruption and fraud
- Health interventions: inefficient mix/inappropriate level of strategies

Source: World health report 2010.^22^

Fourth, the ministry of health can make a stronger case if it can specifically demonstrate that the requested expenditure is part of a coherent investment plan, with resources allocated strategically to where they will achieve the highest impact and value for money. The ministry of finance will expect an accurate and transparent estimate of various costs, including the capital costs, recurrent costs and, most importantly, opportunity costs – that is, what benefits are being foregone if the health ministry’s recommended intervention is adopted, including the cost of doing nothing.^24^ Interventions that simultaneously achieve both efficiency and equity in women’s, children’s and adolescents’ health, as has been the case in Cambodia,^25^ are particularly convincing to a ministry of finance. It also helps their case if health ministry officials can explain, for example, that while the unit costs of expanding antenatal care or immunization to remote and rural areas may be increasing, the cost–effectiveness of those interventions could also be increasing if the burden of disease or risk factors are higher in such areas. Some investment plans fail to apply the lesson that investing in preventive maintenance, for example of a cold-chain supply for immunizations, often yields a much higher economic return than investing in new capital equipment.

Fifth, ministries of finance are usually interested in saving money and reducing costs. Health ministries can therefore help their case if they demonstrate that expenditure on health is not just cost–effective but can also be cost-saving to government and to households. For example, reducing unintended pregnancies through expanded coverage of modern family planning methods is estimated to save US$ 5.1 billion globally that would otherwise be required to provide the recommended care to pregnant women and newborns.^13^ In another example, every 26 days the United States of America saves the total amount of its original contribution to the campaign to eradicate smallpox, because it no longer has to vaccinate against or treat the disease.^26^ Even though many governments currently allocate only relatively small amounts to the health sector, ministries of finance are aware that health-sector expenditure can increase faster than economic growth, inflation and government revenues, and may become financially unsustainable. It is therefore prudent for health ministries to show in their budget submissions that they include cost-saving interventions, when these are warranted on health grounds, and that they are also alert to the need to avoid unproductive and financially unsustainable cost escalation over the longer term.^27^

Sixth, it helps if health ministries can argue when and how market failures in health require public expenditure. Market failures occur when markets do not allocate resources in a way that maximizes overall welfare. In the health sector, this can justify public expenditure, for example on disease surveillance, which the private sector has little commercial incentive to provide, or on communicable diseases, which affect others beyond the immediate patient paying for a service. Well-targeted public expenditure is justified to correct significant market failures, although this does not necessarily mean public provision (that is, government directly providing the service), and this can run the risk of government failures in provision, including waste and inefficiency.^28^ The ministry of finance, however, may still be unwilling to allocate additional resources to the government health sector if this duplicates, or displaces, the existing role of the private sector which, if it is well regulated, is capable of supporting health and social outcomes. Having an up-to-date national health account that maps the sources and uses of health financing from all sectors, including the private sector, enables health ministries to demonstrate to the ministry of finance where, and why, there are gaps in public provision. Cambodia provides a good example of how national health accounts can strengthen the evidence base to drive policy discussions and prioritization and allocation of resources.^29^

Seventh, an effective health funding proposal is one that identifies, and where possible quantifies, where there are mutual gains for both the ministry of finance and the ministry of health. Increasing taxation on tobacco – recently described as the “single best health policy in the world”^30^ – is one example. That is because raising the excise duty on tobacco reduces health risks through reducing consumption while at the same time expanding the financial resources for the ministry of finance (through increased tax revenue) and for the ministry of health (through reduced expenditure on tobacco-related disease).^31^ WHO has estimated that a 50% increase in tobacco excise taxes would generate US$ 1.42 billion in additional funds in the 22 low-income countries for which data are available.^22^ If all of these funds were allocated to health, it would allow government health spending to increase by more than 25% in several countries. Analysis shows that raising excise duties on tobacco is not regressive (that is, disproportionately affecting the poor) over the medium to long term: a claim often made by the tobacco industry lobby and sometimes by finance ministries.^31^ The ministry of health is also in a good position to demonstrate to the finance ministry and other financial decision-makers the mutual benefits to other sectors of investing in women’s, children’s and adolescents’ health: for example, the potential for improved school attendance, educational achievement and worker productivity.

Eighth, presenting a strong plan for the implementation, management and evaluation of health programmes is important. Some ministries of health, and their development partners, place emphasis on the upstream strategic planning but pay less attention to the downstream realities of procurement, health-worker salaries, supply-side readiness and other key aspects of scaling up implementation in practice.^32^^,^^33^ Strategic plans can be derailed during implementation due to subsequent misprocurement or poor budget execution. A good investment plan is one that anticipates possible second-round effects of an intervention. For example, increases in the supply of doctors in a country may lead to increased prescribing of diagnostic tests and drugs that then need to be anticipated and provided for in budgets and supply logistics. In other cases, the funding of building and capital costs of hospitals by development partners may result in a large, and long, tail of recurrent costs, such as staffing, electricity and maintenance, which were not fully anticipated or budgeted for by the ministry of health. Good implementation can be a particular challenge in the aftermath of decentralization of health services, because staff may lack experience in programme and financial management and in managing larger procurement packages.^34^ In general, the poorer the country is, the more important it is to demonstrate that health managers are actively monitoring the use and impact of scarce resources, learning lessons and making necessary adjustments to achieve substantive and sustained results in ways that achieve value for money.

Ninth, strengthening the information and evidence base for policy and programming is important in budget proposals. Ministries of health already collect input-focused data, such as salaries and the number of professional training workshops. Collecting and analysing output and outcome indicators and the incremental costs of scaling up programmes, can better inform policy and programming decisions with the ministry of finance. The health ministry is also in a good position to advocate for and help build the civil registration and vital statistics data that are essential for health and broader national planning. Statistics on births and deaths are incomplete in many countries, with coverage ranging from 50% in Latin America to 25% in south Asia, and a mere 6% in sub-Saharan Africa.^35^ An absence of reliable civil registration and vital statistics data means, for example, that it is not possible to conclude with confidence if a populous country like Nigeria has made progress in achieving the millennium development goal for reducing maternal mortality.^36^

Finally, ministries of health need to be cautious about advocating earmarked (hypothecated) taxes: for example, proposing an increased tax on tobacco and alcohol products for health reasons but asking for the additional revenue be used to fund health-related services such as health promotion. Such taxes do have some justification from a political economy perspective. For example, consumers may be more willing to accept increased taxes on tobacco and alcohol if they perceive the benefits of publicly-funded smoking cessation and alcohol reduction programmes or, in the case of the Philippines, an expanded health insurance programme.^37^ However, ministries of finance tend to be resistant to earmarked taxes, preferring to allocate additional government revenue to the next priority investment area, irrespective of sector. The ministry of finance may well, for example, decide that the extra tax revenue from tobacco and alcohol may have the greatest public benefit when allocated to improving girls’ education, rural road infrastructure, agricultural productivity or electricity generation than if it is allocated to the health sector. Earmarked taxes may, however, be persuasive to a finance ministry official if the health challenge is of the highest national priority: for example, noncommunicable diseases in some Pacific island countries.^38^

## Discussion

Effective action on women’s, children’s and adolescents’ health will always involve adequate and effective public expenditure in the health sector. A key aspect determining this will be the capacity of a health ministry to present coherent investment plans to the ministry of finance. This article has identified 10 key attributes which ministries of finance will normally be looking for when they are assessing requests for financing and which health officials can consider before they start annual budget preparations and negotiations with ministries of finance.

The factors we have identified are sufficiently broad-based that they can be applied in virtually any setting and any country. We recognize, however, that applying these attributes may be difficult in fragile and conflict-affected states, where basic data may be missing and lines of authority and responsibility may be blurred. Furthermore, while many of the attributes also apply to engaging with bilateral and multilateral development partners, other issues may then arise, including the requirement for ministries of health to demonstrate that development partners’ resources are an addition to, rather than a substitution for, government’s own expenditure efforts (what ministry of finance and development partners refer to as fungibility). While we focus on what ministries of health can do, we also recognize that ministries of finance too have a responsibility to improve prioritization, planning and resource allocation. Having a credible ministry of finance medium-term expenditure framework would assist a health ministry to achieve better longer term planning. Releasing funds on time to line departments and allowing the ministry of health more flexibility to transfer resources within budget lines, would improve public expenditure and planning. We also recognize that there are many important contributions to women’s and children’s health that arise as a result of investments and resource allocations to other sectors including for example investments in girls’ education, food security and rural roads.

The Global Strategy for Women’s, Children’s and Adolescents’ Health already provides an overarching policy framework and strategy for improving the health outcomes of women, children and adolescents.^2^ This paper complements this by providing practical suggestions about how health planners can engage more effectively with ministries of finance and other financial decision-makers to access additional funds for the necessary health programmes. There are other published guides on how to make good use of health resources.^24^^,^^39^^–^^44^ Nevertheless, we believe this article goes further, and contributes to the theme of knowledge for effective action on women’s, children’s and adolescents’ health, by explaining how ministry of health officials can anticipate the specific technical arguments and counter-arguments from their own ministry of finance in order to prepare more convincing investment plans.
